# Copper Deficiency Induced Emphysema Is Associated with Focal Adhesion Kinase Inactivation

**DOI:** 10.1371/journal.pone.0030678

**Published:** 2012-01-20

**Authors:** Shiro Mizuno, Masanori Yasuo, Harm J. Bogaard, Donatas Kraskauskas, Aysar Alhussaini, Jose Gomez-Arroyo, Daniela Farkas, Laszlo Farkas, Norbert F. Voelkel

**Affiliations:** 1 Pulmonary and Critical Care Medicine Division and Victoria Johnson Center for Obstructive Lung Diseases, Virginia Commonwealth University, Richmond, Virginia, United States of America; 2 Division of Respiratory Disease, Kanazawa Medical University, Ishikawa, Japan; 3 VU University Medical Center, Amsterdam, The Netherlands; State University of Rio de Janeiro, Biomedical Center, Institute of Biology, Brazil

## Abstract

**Background:**

Copper is an important regulator of hypoxia inducible factor 1 alpha (HIF-1α) dependent vascular endothelial growth factor (VEGF) expression, and is also required for the activity of lysyl oxidase (LOX) to effect matrix protein cross-linking. Cell detachment from the extracellular matrix can induce apoptosis (anoikis) via inactivation of focal adhesion kinase (FAK).

**Methodology:**

To examine the molecular mechanisms whereby copper depletion causes the destruction of the normal alveolar architecture via anoikis, Male Sprague-Dawley rats were fed a copper deficient diet for 6 weeks while being treated with the copper chelator, tetrathiomolybdate. Other groups of rats were treated with the inhibitor of auto-phosphorylation of FAK, 1,2,4,5-benzenetetraamine tetrahydrochloride (1,2,4,5-BT) or FAK small interfering RNA (siRNA).

**Principal Findings:**

Copper depletion caused emphysematous changes, decreased HIF-1α activity, and downregulated VEGF expression in the rat lungs. Cleaved caspase-3, caspase-8 and Bcl-2 interacting mediator of cell death (Bim) expression was increased, and the phosphorylation of FAK was decreased in copper depleted rat lungs. Administration of 1,2,4,5-BT and FAK siRNA caused emphysematous lung destruction associated with increased expression of cleaved capase-3, caspase-8 and Bim.

**Conclusions:**

These data indicate that copper-dependent mechanisms contribute to the pathogenesis of emphysema, which may be associated with decreased HIF-1α and FAK activity in the lung.

## Introduction

Chronic obstructive pulmonary disease (COPD) and emphysema are large global health problems with a high disease prevalence in smokers and individuals exposed to biomass fuel smoke. The pathobiological concepts of COPD/emphysema take genetic and epigenetic risk factors into account and the modern concept of molecular disease mechanisms have recently been reviewed [1]. A postulated lung structure maintenance program [2] provides a large enough conceptual framework for the integration of inherited and acquired mechanisms which can disturb the homeostatic balance which generally preserves the lung function even in individuals at an advanced age. A large body of experimental data characterizes the lung tissue destruction after varying periods of cigarette smoke exposure, however, few studies have addressed mechanisms of lung tissue destruction in non-smokers or animal models where emphysema occurs independent of smoke exposure [3], [4], and little is known about the role of dietary influences on lung structure maintenance.

Here we investigate the impact of a copper-depleted diet and copper chelation on the integrity of the lung alveolar structures. Our studies have been inspired both by a human disease model, the X chromosome-linked, Menkes disease, which is characterized by an inherited copper transporter gene mutation and neonatal emphysema [5], and an animal model, the Blotchy mouse, which also develops emphysema. Underlying the mouse disorder are mutations in the Atp7a gene (the human homologue in Menkes disease is ATP7A), which encodes a copper-transporting ATPase [6], [7]. Copper, an essential trace metal determines the activity of a number of critically important enzymes, among them: lysyl oxidase (LOX) and Cu-Zn SOD. Based on the many important functions of copper-dependent proteins we postulated that copper deficiency would lead to emphysematous lung tissue destruction, as previously reported in the rat and in the hamster [8], [9]. Several mechanisms for the development of emphysema in copper deficient animals have been proposed, but not examined. Although impaired cross linking of matrix protein as a consequence of LOX inhibition provides a rather intuitive explanation for emphysema development in copper-deficient animals, the published data regarding LOX activity dependent emphysema are controversial. Kida et al. reported that administration of a LOX inhibitor caused emphysematous lesions in young rats [10], while Rubio et al. showed that LOX inhibition by itself did not cause emphysematous lesions in adult rats [11]. Based on our investigations, we propose here a particular form of apoptosis, anoikis, as the mechanism of copper-deficiency emphysema. We show that copper-deficiency emphysema is associated with impaired expression of hypoxia inducible factor 1 alpha (HIF-1α) target genes because of impaired HIF-1α transactivation, and we further show that copper deficiency causes a decreased expression and activity of the focal adhesion kinase (FAK).

Taken together, the data derived from our animal model studies establish anoikis as a cause of emphysematous lung tissue destruction and also that proper function of FAK is required for the maintenance of the lung structure.

## Results

### Copper depletion cause emphysema and lung cell apoptosis

**Figure 1:**
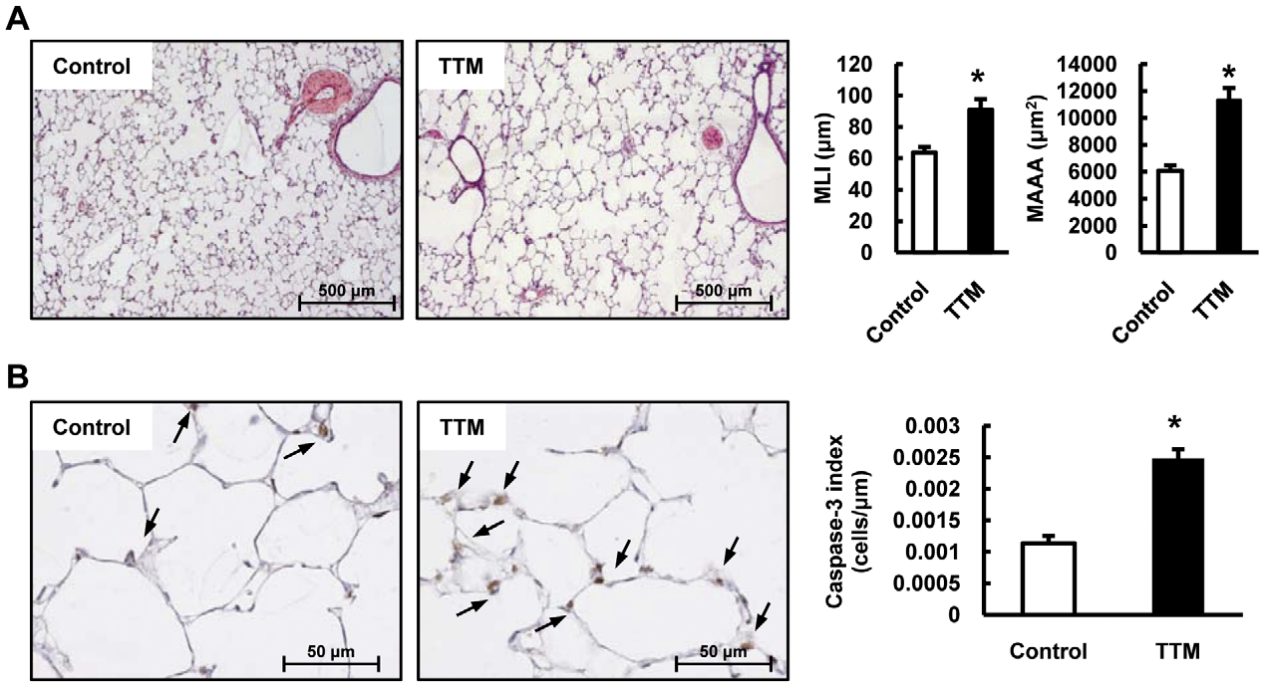
Copper depletion by a copper chelator (tetrathiomolybdate:TTM) treatment causes emphysema and lung cell apoptosis. (A) Representative photomicrographs of HE-stained lung sections of control and TTM treated rats. The bar graph shows the quantification of mean linear intercepts (MLI) and the mean alveolar airspace area (MAAA) in lung sections from Control and TTM treated rats. Data are expressed as mean ± SE (n = 6). *P<0.05 versus control. (B) The photomicrographs of the lung immunohistochemical staining for cleaved caspase-3 are represents of Control and TTM treated rats. The bar graph shows the Caspase-3 positivity index of lung sections from control and TTM treated rats. The Caspase-3 positivity index was calculated as described in the Materials and Methods section. Data are expressed as mean ± SE (n = 6). *P<0.05 versus control.

**Figure 2:**
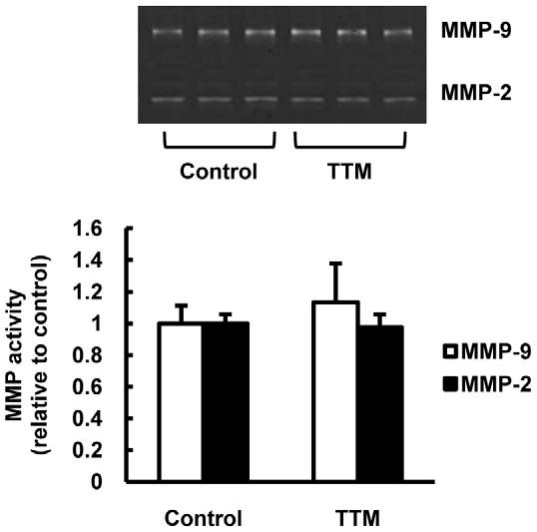
Expression of the activity of MMP-2 and MMP-9 in copper depleted rat lungs. Representative gelatin zymogram of protein extracts from control and TTM treated rat lungs. The bar graph shows the relative density of MMP-2 and MMP-9 bands of TTM treated rat lungs compared to that of control bands. Data are expressed as mean ± SE (n = 6).

We examined rat lung tissue sections stained with hematoxylin and eosin and found that copper depletion caused a significant emphysematous destruction of the lungs, which was uniformly present throughout the tetrathiomolybdate (TTM) treated rat lungs, and an increase of the mean alveolar airspace areas (MAAA) and mean linear intercept (MLI), compared to the control rat lungs (Figure 1A). By examining caspase-3 expression in the rat lung tissues, we found that the airspace enlargement was accompanied by caspase-3 expression (Figure 1B). Emphysematous lung tissue destruction is frequently attributed to activation of proteolysis and in particular to Matrix metalloproteinase (MMP) activation, yet our data do not support a role for MMP-2 and MMP-9 in the emphysema development in the copper depleted rat (Figure 2).

### Altered gene and protein expression in emphysematous lungs

**Figure 3:**
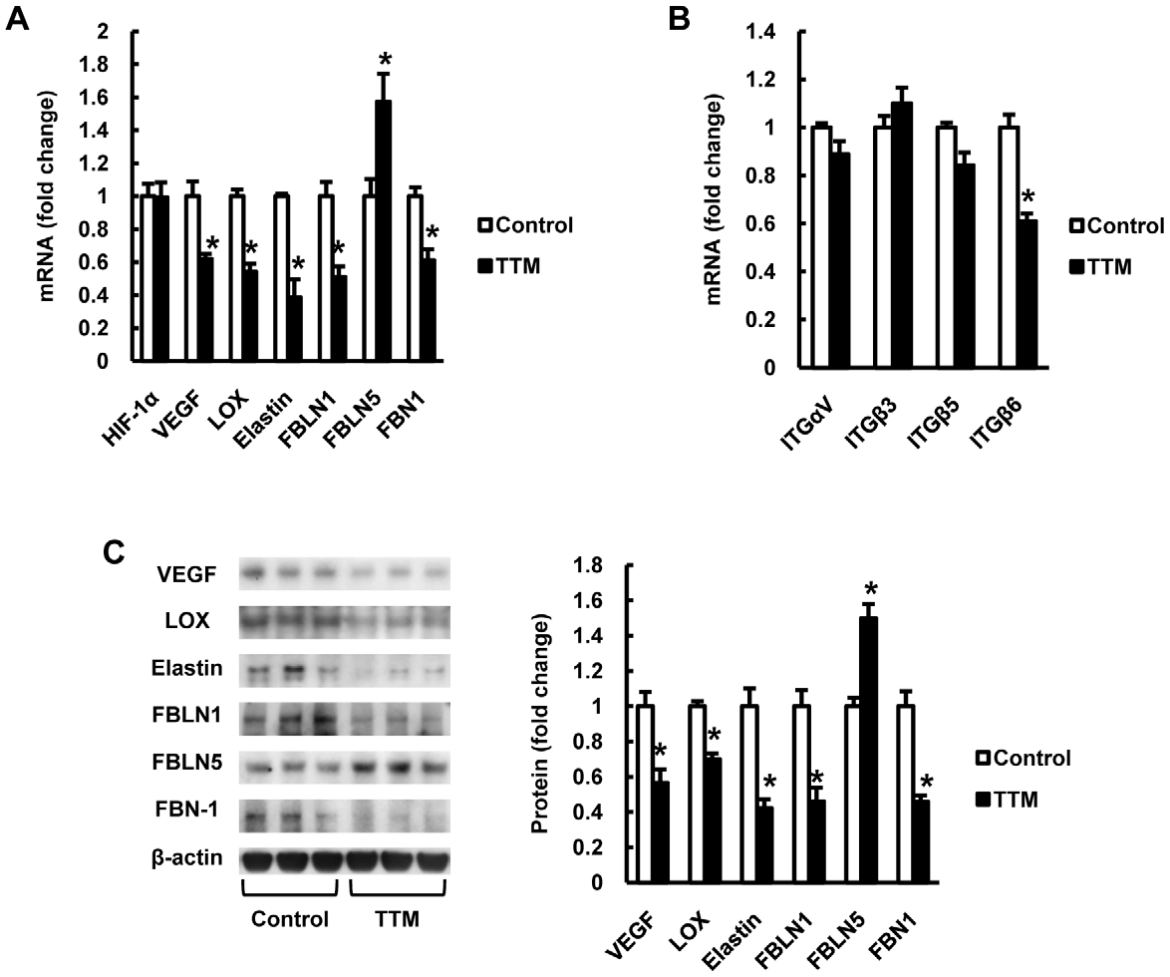
Copper depletion decreases VEGF, lysyl oxidase (LOX), elastin, fibullin-1 (FBLN1), fibrillin-1 (FBN1) and integrin β6 expression in rat lungs. (A) The bar graphs show HIF-1a, VEGF, LOX, elastin, FBLN1, FBLN5 and FBN1 mRNA expression in TTM treated rat lungs measured by RT-PCR analysis, and quantified as a ratio of the expression in vehicle treated rats. (B) The bar graphs show integrin (ITG) αV, β3, β5 and β6 mRNA expression in TTM treated rat lungs.(C) Representative Western blot analysis of VEGF, LOX, FBLN1, FBLN5, FBN1 and ß-actin in lung protein extracts from TTM treated rats. The bar graph shows the ratios of VEGF, LOX, FBLN1, FBLN5, FBN1protein expression relative to controls. Data are expressed as mean ± SE (n = 6). *P<0.05 versus control.

We have previously proposed that the endothelial cell growth and maintenance factor vascular endothelial growth factor (VEGF) is critically important for the health of lung microvascular endothelial cells [12], and because experimental strategies to decrease the expression of lung tissue VEGF or inhibit its actions have resulted in emphysema [13], we examined the lung tissue mRNA expression of VEGF from the copper-deficient rats and found decreased VEGF mRNA and protein expression levels of the mRNA. In addition, we found decreased expression of mRNA and protein levels of LOX, elastin, fibulin 1 (FBLN1) and fibrillin 1 (FBN1), whereas the expression of fibulin 5 (FBLN5) was increased (Figure 3A, C). Furthermore, we found decreased expression of integrin β6 mRNA expression in copper depleted rat lungs (Figure 3B).

### Copper chelation affects lung tissue HIF-1α protein expression and its transactivational activity

**Figure 4:**
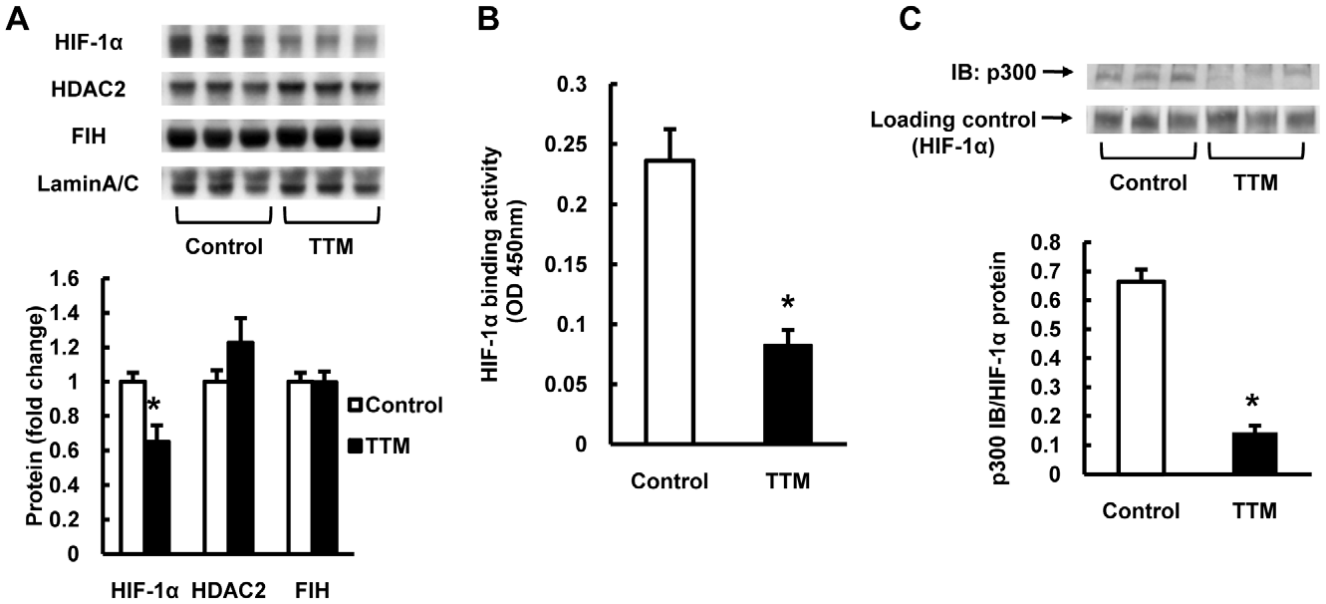
Copper depletion affects lung tissue HIF-1a protein expression and its transactivational activity. (A) Representative Western blot analysis of HIF-1α, histone deacetylase 2 (HDAC2), p300, factor inhibiting HIF (FIH) and Lamin A/C in lung protein extracts from TTM treated rats. The bar graph shows the ratios of HIF-1α, HDAC2, p300 and FIH protein expression relative to controls. (B) HIF-1α activity was measured using a HIF-1α transcriptional factor assay kit and nuclear protein extracts from rat lungs. The activity was decreased in TTM treated rat lungs. (C) Representative photomicrograph of immunoprecipitation of HIF-1α and p300, showing impaired HIF-1α/p300 binding (IB:p300) in TTM treated rat lung nuclear protein extracts. The bar graph shows HIF-1α/p300 binding protein expression relative to the loaded HIF-1α protein. Data are expressed as mean ± SE (n = 6). *P<0.05 versus control.

Because both VEGF and LOX are HIF-1α-dependently transcribed, we examined in nuclear extracts the expression of HIF-1a protein and the expression of three nuclear proteins histone deacetylase 2 (HDAC2), p300 and factor inhibiting HIF (FIH). Whereas HIF-1α protein expression was decreased by copper depletion, the expression of the p300/HAT (histone acetyl transferase) protein was increased by copper depletion (Figure 4A). To assess whether impaired HIF-1α binding to target genes could explain the reduced expression of VEGF and LOX in the lungs from copper depleted rats, the binding of HIF-1α extracted from the lung nuclear protein fraction to hypoxia responsive element (HRE) sequence expressing DNA was assessed. The results obtained using this binding assay indicate impaired lung tissue HIF-1α nuclear protein binding activity in the copper depleted lungs (Figure 4B). Because copper depletion caused in the lung tissue an increased expression of the HIF-1α coactivator p300, we addressed the possibility of impaired HIF-1α/p300 binding. Indeed, immunoprecipitation showed impaired HIF-1α/p300 binding (Figure 4C). Thus we present two separate- but likely linked- causes of impaired (by copper depletion) transactivation of HIF-1α target genes: decreased HIF-1α protein expression and impaired binding of the coactivating protein p300.

### Copper chelation induces the expression of molecular markers which are associated with anoikis

**Figure 5:**
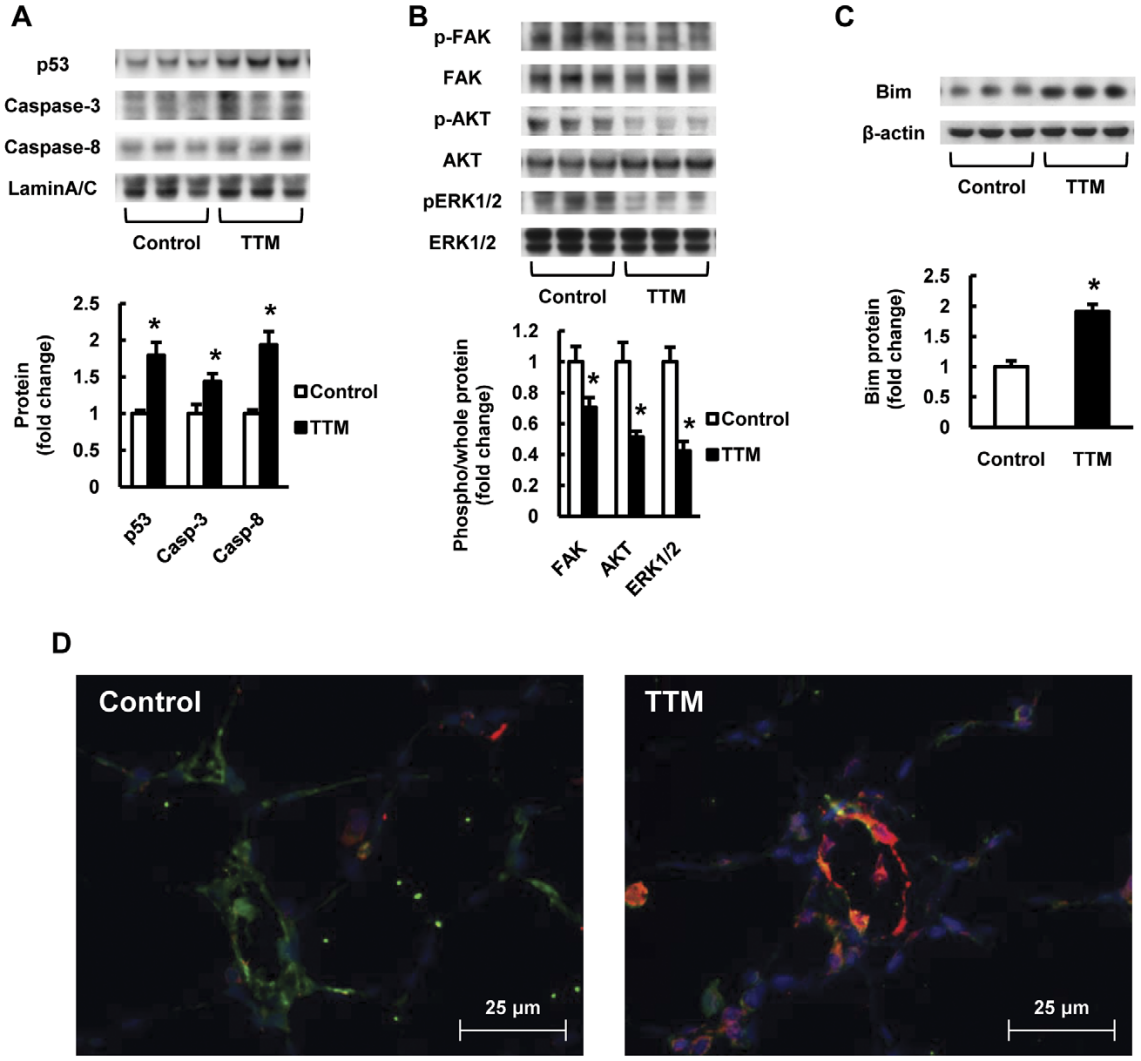
Copper depletion induces anoikis related proteins and inactivates focal adhesion kinase (FAK), AKT and ERK signaling in rat lungs. (A) Representative Western blot analysis of p53, caspase-3, caspase-8 and Lamin A/C in lung protein extracts from TTM treated rats. The bar graph shows the ratios of protein expression relative to Lamin A/C protein. (B) Representative Western blot analysis of phospho-FAK, FAK, phospho-AKT, AKT, phospho-ERK1/2 and ERK1/2 in lung protein extracts from TTM treated rats. The upper bar graph shows the ratios of phosphorylated protein expression relative to each whole protein expression. (C) Representative Western blot analysis of Bim and β-actin in lung protein extracts from TTM treated rats. The bar graph shows the ratios of protein expression relative to control. Data are expressed as mean ± SE (n = 6). *P<0.05 versus control. (D) Dual staining for Bim (red) and von Willebrand factor (vWF) (green) shows that the Bim positive cells are identified in and around the small pulmonary arteries from copper depleted rat lungs. The blue color shows 4,6-Diamidino-2-phenylindole (DAPI) staining.

We found that p53 expression was increased in copper depleted lung tissues (Figure 5A) as in human emphysematous lungs [14]. Based on the combination of copper depletion-induced tissue gene and protein expression changes (decrease in the expression of VEGF and LOX), we considered that a separation of alveolar structure cells from their matrix may occur after copper depletion – resulting in anoikis, loss of cell anchorage-dependent cell death. Therefore, we sought evidence in support of this hypothesis by examining the lung tissue for the expression of protein markers which characterize cells which die by anoikis [15], [16], [17]. Taken together, the protein expression pattern shown in Figure 5B supports the concept that copper depletion induces anoikis in lung alveolar cells, as reduced FAK phosphorylation, reduced AKT and ERK1/2 phosphorylation, induced expression of caspase-8 and of p53 all have been described as markers of anoikis. In addition, we confirmed increased expression of Bim in lungs from copper depleted rats (Figure 5C). Importantly, double staining of Bcl-2 interacting mediator of cell death (Bim) and von Willebrand factor (vWF) shows that increased expression of Bim was found in the cells in and around the small arteries from copper depleted rat lungs (Figure 5D).

### Inhibition of FAK induces emphysema

**Figure 6:**
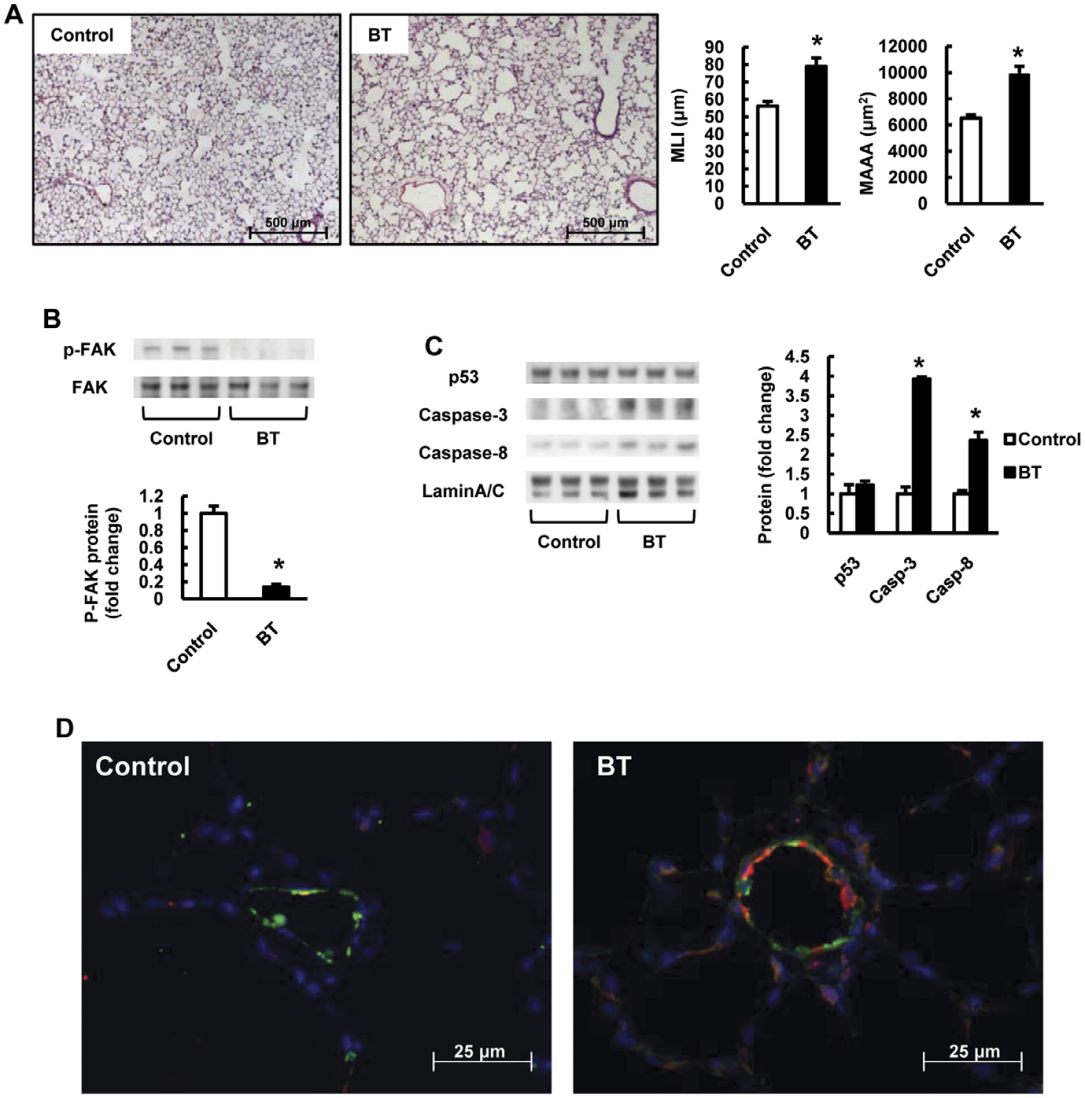
Inhibition of FAK phosphorylation causes emphysema and increases anoikis related protein expressions in rat lungs. (A) Representative photomicrographs of HE-stained lung sections of control and 1,2,4,5-benzenetetraamine tetrahydrochloride (BT) treated rats. The bar graph shows quantification of MLI and MAAA in lung sections from Control and BT treated rats. (B) Representative Western blot analysis of phospho-FAK at Y397 site and FAK in lung protein extracts from BT treated rats. The bar graph shows the ratios of phosphorylated protein expression relative to whole protein expression. (C) Representative Western blot analysis of p53, caspase-3, caspase-8 and Lamin A/C in lung nuclear protein extracts from BT treated rats. The bar graph shows the ratios of the protein expressions relative to control. Data are expressed as mean ± SE (n = 6). *P<0.05 versus control. (D) Dual staining for Bim (red) and von Willebrand factor (vWF) (green) shows that the Bim positive cells are identified in and around the small pulmonary arteries from BT treated rat lungs. The blue color shows DAPI staining.

**Figure 7:**
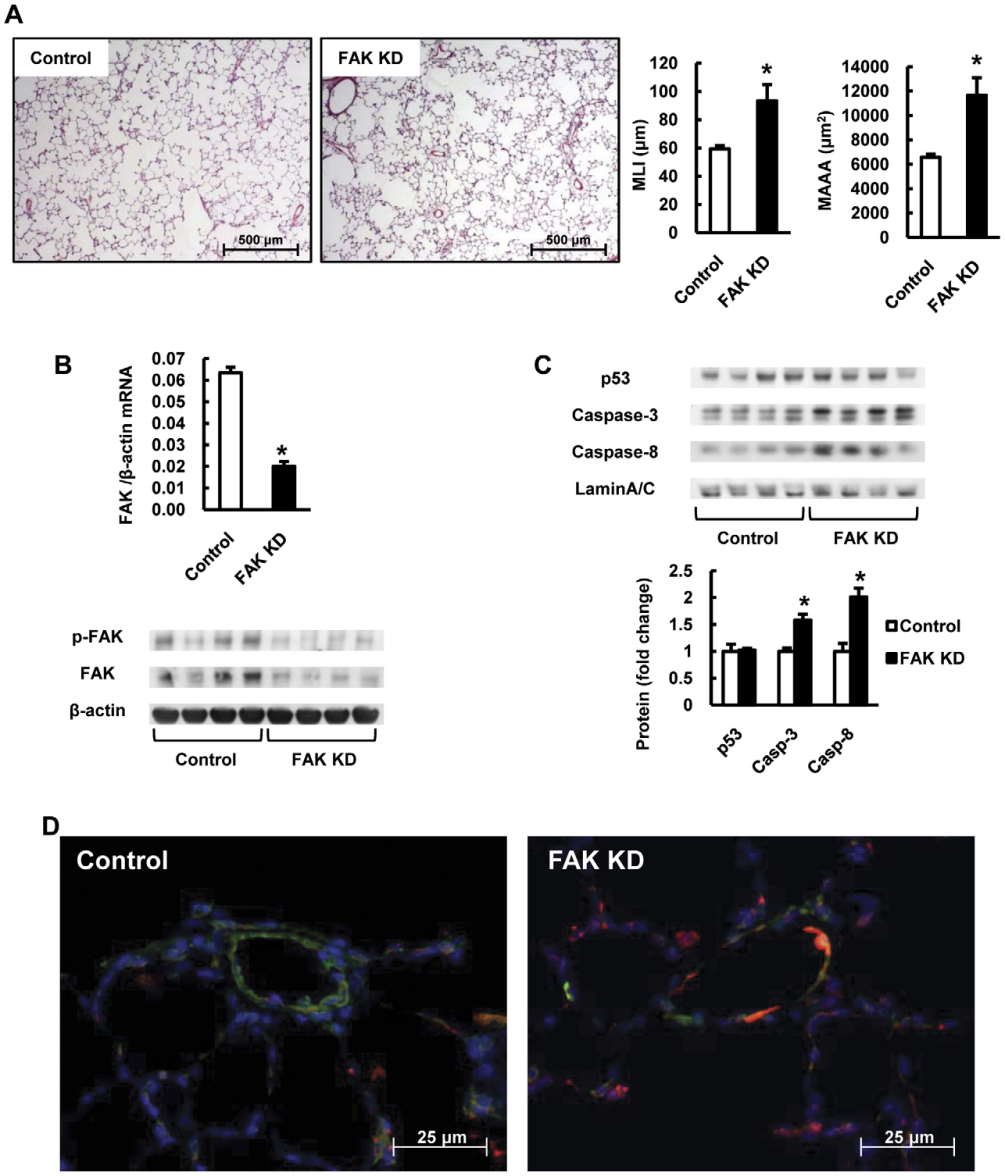
Invivo gene silencing of FAK causes emphysema and increases anoikis related protein expressions in rat lungs. (A) Representative photomicrographs of HE-stained lung sections of control and FAK siRNA treated rats. The bar graph shows quantification of MLI and MAAA in lung sections from control and FAK siRNA treated rats (FAK KD). (B) The bar graph shows the ratios of FAK mRNA expression relative to β-actin measured by RT-PCR. Representative Western blot analysis of phospho-FAK at Y397 site, FAK and β-actin in lung protein extracts from control and FAK siRNA treated rats. (C) Representative Western blot analysis of p53, caspase-3, caspase-8 and Lamin A/C in lung nuclear protein extracts from control and FAK siRNA treated rats. The bar graph shows the ratios of the protein expressions relative to control. Data are expressed as mean ± SE (n = 6). *P<0.05 versus control. (D) Dual staining for Bim (red) and von Willebrand factor (vWF) (green) shows that the Bim positive cells are identified in and around the small pulmonary arteries from FAK siRNA treated (FAK KD) rat lungs. The blue color shows DAPI staining.

Because copper depletion reduced phospho-FAK levels in lung tissues (Figure 5B), consistent with the interpretation that alveolar structure cells undergo anoikis, we next tested whether an inhibitor of FAK phoshorylation or gene silencing of FAK would induce emphysematous lung destruction and anoikis in rat lungs. Indeed, we show that the compound 1,2,4,5-benzenetetraamine tetrahydrochloride (1,2,4,5-BT), known as an inhibitor of auto-phosphorylation of FAK at Y397 [18] and instillation of FAK siRNA caused emphysematous lung destruction (Figure 6A and 7A). The phosphorylation of FAK was decreased both in the 1,2,4,5-BT treated and FAK silenced rat lungs compared with control rat lungs (Figure 6B and 7B). We found increased expression of cleaved caspase-3 and caspase-8 both in the 1,2,4,5-BT treated and FAK silenced rat lungs compared with control rat lungs (Figure 6C and 7C), and increased expression of Bim in and around the small pulmonary arteries (Figure 6D and 7D). In the aggregate, these data support the hypothesis that inhibition of FAK-dependent cell signaling induces anoikis of alveolar septal cells in the lung.

## Discussion

Although copper deficiency has been, both in the human Menkes disease and in the animal model of the Blotchy mouse, firmly linked to emphysema development [5], [7], mechanisms leading to emphysematous lung tissue destruction have not been elucidated in any detail. Here we present data which illustrate how copper deficiency can fundamentally derange transcriptional mechanisms which lead to decreased expression of genes encoding enzymes, growth factors and matrix proteins. Because these disregulations in their combination provide plausible explanations for copper-deficiency induced emphysema, the proteins and signal transduction steps involved in emphysemagenesis can perhaps also be identified as candidate components of the lung structure maintenance program [1], [2]. Here we show for the first time that 1) copper deficiency induces anchorage-dependent cell death, a process important for the maintenance of tissue homeostasis [15] and metastatic cell dissemination in cancers [16], and 2) that the activity of the FAK is necessary to maintain a normal lung structure. The key findings resulting from our experiments are derived mainly from the analysis of lung tissue samples rather from in vitro studies of cultured cells. This allows the observation of the effects of copper depletion in the context of cell-cell and cell-matrix interactions.

We found evidence for lung cell apoptosis in copper depleted rat lungs and also a lack of MMP-2 and MMP-9 activation. Given the absence of inflammatory cell infiltration in the copper-depleted rat lungs, we next turned to the question whether copper depletion affected the expression of the important lung tissue structure maintenance factor VEGF [13]. VEGF gene expression was reduced in the lung tissues from the copper depleted rats. One explanation for the reduced VEGF gene and protein expression was impaired transcription of this HIF-1a target gene [14], in particular because Martin et al. have shown that the prolylhydroxylase (PHD)-dependent HIF-1α protein stability was copper dependent [19]. Because lung tissue HIF-1α protein abundance was only diminished to some degree, we investigated the effect of copper deficiency on lung tissue HIF-1α transactivation activity (binding to HRE). The binding of the nuclear HIF-1α to the HRE was significantly impaired although the expression of the transcriptional co-activator p300 was increased. We therefore immunoprecipitated HIF-1α and p300 protein and found a lack of p300/HIF-1α binding. Feng et al. showed that copper depletion suppresses HIF-1α transcriptional activity in HepG2 cells by inhibition of HIF-1 binding to p300, probably through the inhibition of FIH activity [20]. Our data demonstrating the lack or inhibition of p300/HIF-1α binding (induced by copper depletion) appear to be sufficient to explain the impaired transcriptional activity of HIF-1α and also the reduced expression of LOX. We found that copper depletion also reduced lung tissue FBLN1 gene and protein expression, and such impairment may be associated with severely impaired lung development [21]. Copper depletion also reduced the expression of FBN1. A mutation in the FBN1 gene has been reported in neonatal Marfan syndrome associated with emphysema [22]. Furthermore, we found that elastin gene expression was also reduced in the copper depleted rat lung tissue. This finding complements a previous report based on the examination of fibroblasts from patients with Menkes syndrome [23].

FAK is a cytoplasmic tyrosine kinase that discretely localizes to membrane regions that attach to the extracellular matrix (ECM). FAK transmits signals from the ECM via integrins to the cytoskeleton and in particular to cytoplasmic proteins [15]. Stimulation of integrins and FAK leads to a hypertrophic response in cardiomyocytes [24], and cell to ECM interactions perform critical functions in the control of cell proliferation and survival, [25]. Our data showing a decreased expression of ECM and integrin in copper depleted rat lungs and the association decreased pFAK expression may explain the emphysematous tissue destruction as a consequence of lung cell apoptosis.

The lung tissue samples were also examined for the presence of a protein expression signature reported to occur in cells undergoing anoikis, which includes a reduction in FAK activity. FAK is a scaffold protein that sequesters proapoptotic proteins, such as p53 to mediate cell survival, and a non-receptor tyrosine kinase, which links signals initiated by the extracellular matrix and soluble signaling factors, and FAK is also involved in angiogenesis [26]. For example, it has been shown that reduced expression of FAK reduces the expression of HIF-1α and VEGF in retinal pigment cells [27]. Of importance, hydrogen peroxide caused a marked decrease in the levels of phospho-FAK in mesenchymal stem cells [28], and beta-aminopropionitrile induced inhibition of LOX activation, which is copper dependent, decreased activated FAK in breast cancer cells [29]. Here, we show in the copper depleted lungs a reduction of phospho-FAK, together with increased expression of caspase-8 and both cytosolic and nuclear p53, a pattern consistent with anoikis [15]. Taken together, the hypothesis of copper depletion-induced lung cell death via anoikis is also supported by the reduced tissue expression of phospho-AKT and phospho-ERK1/2 and increased expression of Bim, which is known as an important mediator of anoikis [30].

Because FAK activity may command a central place in the lung structure maintenance program, we treated rats with an inhibitor of FAK phosphorylation and FAK siRNA, and found that FAK inhibition caused both anoikis and emphysema (Figure 6A and 7A). It is known that FAK regulates cardiomyocyte survival [31] and that cardiac FAK knockout causes eccentric right ventricular hypertrophy [32]. We now suggest that impaired FAK activation results in emphysema, since inhibition of FAK caused emphysema and generated a lung tissue expression pattern which is consistent with anoikis.

We found that inactivation of FAK is associated with the development of emphysematous lesions in rat lungs. Our data have been derived from histological, immunohistochemical and morphometric parameters, but are not independently supported by changes in lung function. Compliance measurement of the lungs could be performed in future studies to functionally describe the emphysematogenesis which is caused by copper deficiency.

In conclusion, we have attempted to elucidate molecular mechanisms of copper deficiency-induced emphysema and propose anoikis as a previously not described form of lung cell death. Copper deficiency-induced lung cell anoikis could be an epigenetic cause of emphysema formation in smokers and non smokers.

## Materials and Methods

### Ethics Statement

The protocol was approved by the animal care and use committee (IACUC) of the Virginia Commonwealth University. Our VCU (Virginia Commonwealth University) IACUC protocol number for the copper depletion studies is: AM 10162.

### Animal Experimental Protocols

Male Sprague-Dawley rats (4 weeks old) received a copper-deficient diet (TD 80388, Harlan Laboratories, Inc. Indianapolis, IN) and were injected intraperitoneally with 10mg/kg body weight of TTM once a day for the first 2 weeks. Control rats received a normal diet (7012 Teklad LM-485 Mouse/Rat Sterilizable Diet, Harlan Laboratories, Inc.) and were injected with PBS only. Other groups of male Sprague-Dawley rats (4 weeks old) received 30 mg/kg BW of the inhibitor of FAK phosphorylation, 1,2,4,5-benzenetetraamine tetrahydrochloride 5 days/week, intraperitoneally. Control rats received PBS only. After 6 weeks, each rat was anesthetized with an intraperitoneal injection of thiobarbital sodium. The thoracic cavities were opened by midline incision, and a small sample of blood was obtained by cardiac puncture and placed in a heparinized tube for hematocrit determination. Right lungs were removed, and placed into RNAlater (Ambion) or frozen in liquid nitrogen. Left lungs were inflated with 0.5% low-melting agarose at a constant pressure of 25 cm H_2_O, fixed in 10% formalin for 48 hours and used for morphometry and immunohistochemical analysis.

### Invivo transfection of FAK siRNA in rats

FAK and negative control invivo siRNA were designed and synthesized by Invitrogen (Sunnyvale, CA). The FAK a siRNA target sequences were 5′-GGGCCAGUAUUAUCAGGCAUGGAGA-3′and control siRNA target sequences were 5′-AGCAUAGGUUCCAUGUCCAUCAAUA-3′. The mixture of the siRNA and InvivofectamineTM (Invitrogen) reagent complexed with target siRNA were prepared according to the manufacture's protocol. Male Sprague-Dawley rats (4 weeks old) received the complex of Invivofecatmine™ and FAK or control siRNA at a dose of 1.5 ml/kg BW, 2 days/week by way of nasal instillation. Briefly, we restrained the rats following ketamine-induced anesthesia and placed the rat on its back on a warming pad, and carefully injected 1.5 ml/kg of the invivofectamine-siRNA complex (1 mg/ml siRNA) slowly into each nose. After 3 weeks, each rat was sacrificed as described.

### Antibodies

Rabbit anti-cleaved caspase-3 antibody and rabbit anti-Bim antibody (Cell Signaling Technology, Inc., Beverly, MA), rabbit anti-LOX anbibody (Novus Biologicals, Littleton, CO), mouse anti-HIF-1α antibody, rabbit anti-HIF-1α antibody, rabbit anti-p300 antibody, mouse anti-VEGF antibody, rabbit anti- HDAC2 antibody, mouse anti-p53 antibody, mouse anti-caspase-8 antibody, rabbit anti-phosphor-FAK (Y394) antibody, mouse anti-FAK antibody, rabbit anti-phosphor-AKT antibody, mouse anti-AKT antibody, mouse anti-phospho-ERK antibody, rabbit anti-ERK antibody, goat anti-FBLN1 antibody, rabbit anti- FBLN5 antibody, rabbit anti-FBN1 antibody, goat anti-elastin antibody and mouse anti-Lamin A/C antibody (Santa Cruz Biotechnology, Inc., Santa Cruz, CA), and mouse anti-ß-actin antibody (Sigma, St. Louis, MO).

### Morphometry

Lungs were inflated with 0.5% low-melting agarose at a constant pressure of 25 cm H_2_O, fixed in 10% formalin for 48 hours and paraffin-embedded by standard techniques. Sections (3 µm) were stained with hematoxylin and eosin. Images were acquired with a Carl Zeiss AxioCam color camera (Carl Zeiss Vision GmbH, Hallbergmoos, Germany) and analyzed using AxioVision® Imaging System software (Carl Zeiss Vision GmbH). 10 lung fields per tissue section were captured at a 100 x magnification, then AxioVision® Imaging System software was used to measure the MAAA and total length of alveolar perimeters (TLAP), the imaging system was also used to identify and quantify caspase-3 positive cells in lung sections (caspase-3 index), in pixels per µm^2^. Emphysematous changes were also assessed by measurement of the MLI. MLI is a measurement of mean interalveolar septal wall distance, which is widely used to examine alveolar space size. The MLI was measured by dividing the length of a line drawn across the lung section by the total number of intercepts counted within this line, at x 100 magnification. A total of 40 lines per each rat lung were drawn and measured.

### Immunohistochemical staining of cleaved caspase-3 and dual staining of Bim and vWF

For staining of cleaved caspase-3, the slides with 3 µm paraffin sections were deparaffinized in xylene, and rehydrated, and then submitted to microwave treatment in 10 mM citric acid monohydrate solution. After quenching of endogenous peroxidase with 3% H_2_O_2_ for 15 minutes, the slides were incubated overnight with anti-cleaved caspase-3 rabbit polyclonal antibody (1∶ 200 dilution) at 4°C and were subsequently incubated with biotinylated anti-rabbit IgG antibody for 30 min at room temperature. Following this secondary antibody application, sections were incubated with ABC complex (Vector, Burlingame, CA) for 30 min at room temperature, and developed with diaminobenzidine (DAB; Vector, Burlingame, CA) until the desired stain intensity had developed. A light hematoxylin counterstain was applied. 10 lung fields per tissue section were captured at a 400 x magnification, and then the number of active caspase 3-positive cells was counted using the AxioVision® Imaging System software using a color thresh-holding function. In each capture picture, the degree of caspase-3 positivity was indexed for the TLAP. Normal rabbit serum in the absence of primary antibody was used as a negative control.

For double immunofluorescence staining, lung sections (3 µm) were deparaffinized and rehydrated. After antigen retrieval (20 minutes with 0.01 M citrate buffer pH 6.0), slides were blocked with 1% NSS in TBS for 15 minutes. Then, slides were incubated with primary antibody #1 (rabbit anti-Bim,antibody, 1∶200) at 4°C overnight. Anti- rabbit Alexa Fluor 488 1∶100 was applied for 4 hours at room temperature, then slides were incubated with primary antibody #2 (vWF, 1∶25 LifeSpan BioSciences) overnight at 4°C. Then, anti- mouse Dylight 594 1∶200 (JacksonImmunoResearch) was applied for 4 hours at room temperature. Slides were counterstained with 4′,6-diamidino-2-phenylindole (DAPI) 1∶20 000 for 5 minutes and coverslipped using SlowFade® Antifade (both Invitrogen). Negative controls with nonspecific IgG were run in parallel. Images were acquired with an IX70 microscope and MagnaFire 1.1 software (both Olympus) using a 40x objective (400x magnification). Color composite images were generated with ImageJ software (Rasband, W.S., ImageJ, U. S. National Institutes of Health, Bethesda, Maryland, USA, http://imagej.nih.gov/ij/, 1997-2011).

### Gelatin Zymography

MMP-2 and MMP-9 activity were determined by gelatin zymography. Protein extracts (25 µg) were electrophoresed on 10% SDS-Tris-glycine gels containing 1 mg/ml gelatin (Invitrogen, Sunnyvale, CA). The gels were re-natured, developed, and stained with Comassie brilliant blue and destained as per standard protocol (Bio-Rad, Hercules, CA). Proteolysis bands were visualized and quantified using Printgraph.

### HIF-1α activity assay

HIF-1α transcriptional activity in lung nuclear extracts lung were measured using a HIF-1α transcriptional factor assay kit (Cayman, Ann Arbor, MI) according to the manufacture's protocol.

### Real-time RT-PCR analysis of mRNA using LightCycler480™

Isolation of total RNA from lung was performed using a miRNeasy Mini kit (Qiagen, Valencia, CA) according to the manufacture's protocol. Total RNA (1 µg) was reverse-transcribed using random primer and MultiScribe RT (High-Capacity cDNA Archive Kit, Applied Biosystems, Foster City, CA) for mRNA analysis. Polymerase chain reaction (PCR) was performed with the resulting reverse transcription products using specific oligonucleotide primers. The sequence of these primers is shown in Table 1.

**Table 1:** Primer sequences used for real-time quantitative PCR.

Target gene	Sense-primer	Antisense-primer
HIF-1α	5′-AAGTCAGCAACGTGGAAGGT-3′	5′-CGTCATAGGCGGTTTCTTGT-3′
VEGF	5′-TCCTCTCCCTACCCCACTTC-3′	5′-AAGCCACTCACACACACAGC-3′
LOX	5′-ACTCCGACGACAACCCCTAT-3′	5′-ACGTGGATGCCTGGATGTAG-3′
Elastin	5′-CTTGCTCAACCTCCTCCATC-3′	5′-CTCCTCCGATACCAGCTCCT-3′
FBLN1	5′-GGAGGAGGAACAAGAAGACC-3′	5′-CACAAAGCAAGAGCAGATGA-3′
FBLN5	5’-GTGTGTGGATGTGGACGAGT-3′	5′-TACCCTCCTTCCGTGTTGAT-3′
FBN1	5′-TTGCCAGAATACACCAGGAA-3′	5′-GTTACCCTCACACTCGTCCA-3′
ITGαV	5′-CGTTTCTATCCCACCACAGG-3′	5′-GCAGTTGAGTTCCAGCCTTC-3′
ITGβ3	5′-TATCCTGGTGGTCCTGCTGT-3′	5′-CTCTGGCTCGTTCTTCCTCA-3’
ITGβ5	5′-GGTTTCGGGTCTTTTGTTGA-3′	5′-GGGACACAGTTGGGGAATAA-3′
ITGβ6	5′-GAAAAAGACTGCCCCAAACC-3′	5′-CAGTAGCACGACACCGATGA-3′
FAK	5′-AAATTGTCCTCCCACCCTCT-3′	5′-TCCATCCTCATCCGTTCTTC-3′
ß-actin	5′-CCGTGAAAAGATGACCCAGA-3′	5′-GAGGCATACAGGGACAACAC-3′

All PCR reactions were performed with a LightCycler480™ PCR system (Roche Diagnostics, Meylan, France) using DNA binding SYBR Green dye (Applied Biosistems) for mRNA analysis for the detection of PCR products. The cycling conditions were as follows: initial denaturation at 95°C for 15 minutes, followed by 50 cycles of denaturation at 94°C for 15 seconds, annealing at 57°C for 15 seconds, and extension at 72°C for 15 seconds. The ß-actin gene was used as the reference of mRNA. The PCR products were visualized by electrophoresis on 1.5% agarose gels with ethidium bromide staining to confirm the products.

### Western blot analysis

Cytoplasmic and nuclear proteins from lungs and cells were prepared using NE-PER Nuclear and Cytoplasmic Extraction Reagents based on the manufacturer's protocol (Pierce, Rockford, IL), and the protein extracts were analyzed for protein content using a Bradford method. Each sample was quantified, and then 40 µg of cytoplasmic protein or 20 µg of nuclear protein was loaded into each lane of a 4–12% Bis-Tris Nupage gel with MES SDS running buffer, according to the manufacturer's protocol. The gel was transferred to a PVDF membrane by electrophoresis at 100 V for 1 hour. The membrane was blocked in PBS, 0.2% Tween 20 (PBS-T), and 5% nonfat milk at room temperature for 1 hour. All antibodies were diluted in the same blocking buffer. The membrane was then probed with the primary antibodies. Subsequently, membranes were incubated with horseradish peroxidase-conjugated goat anti-mouse, goat anti-rabbit, or chicken anti-goat antibody. The ECL system was used for detection of the proteins. Each assay was performed in 6 independent experiments.

### Immunoprecipitation

Immunoprecipitation was performed using mouse anti-HIF1α antibody and ExactaCruz™ Immunoprecipitation kit (Santa Cruz Biotechnology) according to the manufacture's protocol. The immunoprecipitates were subjected to Western blotting analysis as described above and HIF-1α/p300 binding proteins were visualized using rabbit anti-p300 antibody and ECL system. The loaded HIF-1α proteins were visualized using a rabbit anti-HIF-1α antibody.

### Statistical Analysis

Results are expressed as mean ± SE. Statistical analysis was performed using ANOVA with Bonferroni corrections for multiple comparisons. Comparisons were considered statistically significant at p<0.05.

## References

[1] Taraseviciene-Stewart L, Voelkel NF (2008). Molecular pathogenesis of emphysema.. J Clin Invest.

[2] Tuder RM, Yoshida T, Fijalkowka I, Biswal S, Petrache I (2006). Role of lung maintenance program in the heterogeneity of lung destruction in emphysema.. Proc Am Thorac Soc.

[3] Salvi SS, Barnes PJ (2009). Chronic obstructive pulmonary disease in non-smokers.. Lancet.

[4] Behrendt CE (2005). Mild and moderate-to-severe COPD in nonsmokers: distinct demographic profiles.. Chest.

[5] Grange DK, Kaler SG, Albers GM, Petterchak JA, Thorpe CM (2005). Severe bilateral panlobular emphysema and pulmonary arterial hypoplasia: unusual manifestations of Menkes disease.. Am J Med Genet A.

[6] Hunt DM (1974). Primary defect in copper transport underlies mottled mutants in the mouse.. Nature.

[7] Ranga V, Grahn D, Journey TM (1983). Morphologic and phenotypic analysis of an outcross line of blotchy mouse.. Exp Lung Res.

[8] Soskel NT, Watanabe S, Sandberg LB (1984). Mechanisms of lung injury in the copper deficient hamster model of emphysema.. Chest.

[9] O'Dell BL, Kilburn KH, McKenzie WN, Thurston RJ (1978). The lung of the copper-deficient rat. A model for developmental pulmonary emphysema.. Am J Pathol.

[10] Kida K, Thurlbeck WM (1980). The effects of beta-aminopropionitrile on the growing rat lung.. Am J Pathol.

[11] Rubio ML, Sánchez-Cifuentes MV, Peces-Barba G, Verbanck S, Paiva M (1998). Intrapulmonary gas mixing in panacinar- and centriacinar-induced emphysema in rats.. Am J Respir Crit Care Med.

[12] Kasahara Y, Tuder RM, Cool CD, Lynch DA, Flores SC (2001). Endothelial cell death and decreased expression of vascular endothelial growth factor and vascular endothelial growth factor receptor 2 in emphysema.. Am J Respir Crit Care Med.

[13] Kasahara Y, Tuder RM, Taraseviciene-Stewart L, Le Cras TD, Abman S (2000). Inhibition of VEGF receptors causes lung cell apoptosis and emphysema.. J Clin Invest.

[14] Yasuo M, Mizuno S, Kraskauskas D, Bogaard HJ, Natarajan R (2011). Hypoxia inducible factor-1a in human emphysema lung tissue.. Eur Respir J.

[15] Grossmann J (2002). Molecular mechanisms of “detachment-induced apoptosis—Anoikis”.. Apoptosis.

[16] Stupack DG, Teitz T, Potter MD, Mikolon D, Houghton PJ (2006). Potentiation of neuroblastoma metastasis by loss of caspase-8.. Nature.

[17] Lim ST, Chen XL, Lim Y, Hanson DA, Vo TT (2008). Nuclear FAK promotes cell proliferation and survival through FERM-enhanced p53 degradation.. Mol Cell.

[18] Golubovskaya VM, Nyberg C, Zheng M, Kweh F, Magis A (2008). A small molecule inhibitor, 1,2,4,5-benzenetetraamine tetrahydrochloride, targeting the y397 site of focal adhesion kinase decreases tumor growth.. J Med Chem.

[19] Martin F, Linden T, Katschinski DM, Oehme F, Flamme I (2005). Copper-dependent activation of hypoxia-inducible factor (HIF)-1: implications for ceruloplasmin regulation.. Blood.

[20] Feng W, Ye F, Xue W, Zhou Z, Kang YJ (2009). Copper regulation of hypoxia-inducible factor-1 activity.. Mol Pharmacol.

[21] Kostka G, Giltay R, Bloch W, Addicks K, Timpl R (2001). Perinatal lethality and endothelial cell abnormalities in several vessel compartments of fibulin-1-deficient mice.. Mol Cell Biol.

[22] Shinawi M, Boileau C, Brik R, Mandel H, Bentur L (2005). Splicing mutation in the fibrillin-1 gene associated with neonatal Marfan syndrome and severe pulmonary emphysema with tracheobronchomalacia.. Pediatr Pulmonol.

[23] Gacheru S, McGee C, Uriu-Hare JY, Kosonen T, Packman S (1993). Expression and accumulation of lysyl oxidase, elastin, and type I procollagen in human Menkes and mottled mouse fibroblasts.. Arch Biochem Biophys.

[24] Laser M, Willey CD, Jiang W, Cooper G 4th, Menick DR (2000). Integrin activation and focal complex formation in cardiac hypertrophy.. J Biol Chem.

[25] Bouchard V, Harnois C, Demers MJ, Thibodeau S, Laquerre V (2008). B1 integrin/Fak/Src signaling in intestinal epithelial crypt cell survival: integration of complex regulatory mechanisms.. Apoptosis.

[26] Keledjian K, Garrison JB, Kyprianou N (2005). Doxazosin inhibits human vascular endothelial cell adhesion, migration, and invasion.. J Cell Biochem.

[27] Zhu J, Wang YS, Zhang J, Zhao W, Yang XM (2009). Focal adhesion kinase signaling pathway participates in the formation of choroidal neovascularization and regulates the proliferation and migration of choroidal microvascular endothelial cells by acting through HIF-1 and VEGF expression in RPE cells.. Exp Eye Res.

[28] Song H, Cha MJ, Song BW, Kim IK, Chang W (2010). Reactive oxygen species inhibit adhesion of mesenchymal stem cells implanted into ischemic myocardium via interference of focal adhesion complex.. Stem Cells.

[29] Payne SL, Fogelgren B, Hess AR, Seftor EA, Wiley EL (2005). Lysyl oxidase regulates breast cancer cell migration and adhesion through a hydrogen peroxide-mediated mechanism.. Cancer Res.

[30] Noda T, Iwai S, Hamada M, Fujita Y, Yura Y (2009). Induction of apoptosis of detached oral squamous cell carcinoma cells by safingol.. Possible role of Bim, focal adhesion kinase and endonuclease G. Apoptosis.

[31] Hakim ZS, DiMichele LA, Rojas M, Meredith D, Mack CP (2009). FAK regulates cardiomyocyte survival following ischemia/reperfusion.. J Mol Cell Cardiol;.

[32] Peng X, Wu X, Druso JE, Wei H, Park AY (2008). Cardiac developmental defects and eccentric right ventricular hypertrophy in cardiomyocyte focal adhesion kinase (FAK) conditional knockout mice.. Proc Natl Acad Sci U S A.

